# Increased physical Activity is Associated With Enhanced Development of Peak Bone Mass in Men: A Five-year Longitudinal study

**DOI:** 10.1002/jbmr.1549

**Published:** 2012-01-13

**Authors:** Martin Nilsson, Claes Ohlsson, Anders Odén, Dan Mellström, Mattias Lorentzon

**Affiliations:** 1 Centre for Bone and Arthritis Research, Sahlgrenska Academy, Institute of Medicine, University of GothenburgGothenburg, Sweden; 2 Department of Mathematical Sciences, Chalmers University of TechnologyGothenburg, Sweden

**Keywords:** LONGITUDINAL STUDY, BONE GEOMETRY, PHYSICAL ACTIVITY, MEN, BONE DENSITY

## Abstract

Data supporting physical activity guidelines to optimize bone development in men is sparse. Peak bone mass is believed to be important for the risk of osteoporosis later in life. The objective of this study was to determine if an increased amount of physical activity over a 5-year period was associated with increased bone mineral content (BMC), areal (aBMD) and volumetric (vBMD) bone mineral density, and a favorable development of cortical bone size in young adult men. The original 1068 young men, initially enrolled in the Gothenburg Osteoporosis and Obesity Determinants (GOOD) study, were invited to participate in the longitudinal study, and a total of 833 men (78%), 24.1 ± 0.6 years of age, were included in the 5-year follow-up. A standardized self-administered questionnaire was used to collect information about patterns of physical activity at both the baseline and 5-year follow-up visits. BMC and aBMD were measured using dual energy X-ray absorptiometry, whereas vBMD and bone geometry were measured by peripheral quantitative computed tomography. Increased physical activity between the baseline and follow-up visits was associated with a favorable development in BMC of the total body, and aBMD of the lumbar spine and total hip (*p* < 0.001), as well as with development of a larger cortex (cortical cross sectional area), and a denser trabecular bone of the tibia (*p* < 0.001). In conclusion, increased physical activity was related to an advantageous development of aBMD, trabecular vBMD and cortical bone size, indicating that exercise is important in optimizing peak bone mass in young men. © 2012 American Society for Bone and Mineral Research.

## Introduction

Peak bone mass is believed to be achieved before the end of the third decade in life, depending on bone site, and low peak bone mass has been considered as a risk factor for developing osteoporosis later in life.(1–3) Genetic factors are the strongest determinants of bone mass,(3) but exercise with loading of the bone also has a major impact on bone mass(4–6) as well as on bone strength.(5, 7, 8) Previous intervention studies in children, adolescents, and young adults have reported that physical activity interventions result in increased bone mineral content (BMC), areal bone mineral density (BMD), and cortical bone size.(9, 10) The skeleton in older persons seems to be less adaptive to physical activity induced mechanical loading applied to it.(10, 11) Due to methodological difficulties, no physical activity intervention studies over several years investigating the development of peak bone mass have yet been performed.

A few small longitudinal studies(12, 13) have found that a maintained high level of physical activity was associated with preserved areal bone mineral density (aBMD) in both young adult men(12) and women.(13, 14) However, neither of these studies were able, due to limited statistical power, to investigate whether changed physical activity was related to altered aBMD. To our knowledge, there are no population-based longitudinal studies that have investigated the association between increased level of physical activity and bone development in young adult men.

In the large majority of previous studies investigating the association between exercise and bone mass, bone properties have been measured using dual energy X-ray absorptiometry (DXA). Because the DXA technique cannot distinguish whether changes in aBMD are due to bone volumetric BMD or in bone geometrical parameters,(15) data regarding the role of physical activity on bone structural parameters is scarce.

We have previously reported, in a cross-sectional analysis in the Gothenburg Osteoporosis and Obesity Determinants (GOOD) study, that physical activity was associated with aBMD and cortical bone size in 18- to 20-year-old Swedish men, and that the boys who began their physical activity before puberty had higher adult aBMD and cortical bone size than boys who started training later.(16)

The aim of this large 5-year longitudinal study of young adult men was to determine whether an increased amount of physical activity in young adulthood was associated with a favorable development in aBMD and volumetric BMD (vBMD), and in cortical bone geometry.

## Subjects and Methods

### Subjects

The study was conducted on participants of the GOOD study, a longitudinal population-based study with the aim to determine both environmental and genetic factors involved in the regulation of bone mass.(17) Details of the cohort have previously been described.(17–19) Briefly, we contacted the original 1068 study subjects and invited them to participate in this 5-year follow-up study. A total of 833 men (78%), 24.1 ± 0.6 years of age (mean ± SD), were included in the present study, with an average time of 61.2 ± 2.3 months between the baseline and follow-up visits.(19) The original GOOD cohort was found representative of the general young male population in Gothenburg,(17, 18) and the cohort of the present study was found to be representative of the initially included population.(19) There were no significant differences between the included and not included subjects in age, height, weight, or amount of present physical activity.(19) The study was approved by the regional ethical review board at the University of Gothenburg. Written and oral informed consent was obtained from all study participants.

### Physical activity

At both the first study visit (baseline) and the 5-year follow-up visit, a standardized self-administered questionnaire, based on a validated physical activity questionnaire to measure the relationship between mechanical strain and bone mass(20) with amendments, was used to collect information about patterns of present physical activity at both visits. Information on type as well as of time (h/week) spent on physical activities in relation to sports was collected. At the baseline visit 529 subjects were physically active and 304 did not participate in any physical activity in relation to sports (sedentary), whereas 531 subjects were active at the 5-year follow-up visit and 302 were then sedentary. Sport activity type (strain) was categorized according to strain score, based on ground reaction forces of physical activity, and classified according to a method previously described.(21, 22) Activities involving jumping actions (eg, gymnastics, handball, basket) were given a strain score of 3, activities including explosive actions like turning and sprinting (eg, soccer, tennis, ice hockey) were given a strain score of 2, whereas other weight-bearing activities (eg, jogging, martial arts, strength training) were given a strain score of 1. Nonimpact activities (eg, swimming, bicycling) were given a strain score of 0.(21, 22) In order to analyze the role of both type and time spent on sport activity on bone parameters, we calculated an osteogenic index based upon a previously described method.(23) In this modified version, the osteogenic index was constructed by multiplying the time spent on each type of sport activity (h/week) with the sport activity strain score (strain score 0–3 based upon known ground reaction forces) for each type of sport activity and then summarizing all the products for all types of sport activity for each subject at the baseline visit and at follow-up visit, respectively. Both change in sport activity (h/week) and osteogenic index (h/week × strain score) between visits were used as continuous variables in the main regression analysis.

To illustrate how bone gain was associated with consistency or change in amount of physical activity between the baseline and 5-year follow-up visit, subjects were divided into two groups, high and low physical activity. We previously reported in the original GOOD cohort, that men with physical activity less than 4 h/week did not have higher aBMD or greater cortical bone size than men who did not participate in any physical activity in relation to sports (sedentary).(17) Thus, in the present study, we divided men into groups based on high (H, defined as ≥4 h/week) and low (L, defined as <4 h/week and sedentary) amount of physical activity.(17) Recorded changes in amount of physical activity between the baseline and follow-up visit were then used to divide the subjects into four groups as follows: consistently high (HH, *n* = 146), consistently low (LL, *n* = 405), changed from high to low (HL, *n* = 213), and changed from low to high (LH, *n* = 69). The following types of physical activity were the most common among subjects who were active in sports at the baseline and/or at the follow-up visits (each subject could have participated in several types of sports): strength training, soccer, running/jogging, martial arts, floor ball, handball, bicycling/spinning, tennis, ice hockey, badminton, and swimming/diving.

### Covariates at both the baseline and follow-up visit

Height and weight were measured using standardized equipment. The coefficient of variation (CV) values were <1% for these measurements. A standardized self administered questionnaire was used to collect information about calcium intake and smoking (yes/no). Calcium intake (mg/day) was estimated from dairy product intake.

### aBMD

We assessed BMC (g) of the total body and aBMD (g/cm^2^) of the lumbar spine (L1–L4), total hip, and nondominant radius using a DXA device (Lunar Prodigy DXA; GE Lunar, Madison, WI, USA). The CV for the aBMD measurements ranged from 0.5% to 3%, depending on application. Five subjects could not undergo total body, lumbar spine, or total hip scan due to weight limits of the Lunar Prodigy DXA.(19)

### Cortical bone geometry and volumetric BMD

We used a peripheral quantitative computed tomography (pQCT) device (XCT-2000; Stratec Medizintechnik, Pforzheim, Germany) to scan the distal leg (tibia) and the distal arm (radius) of the nondominant leg and arm, respectively. A 2-mm-thick single tomographic slice was scanned with a voxel size of 0.50 mm. The cortical vBMD (not including the bone marrow; mg/cm^3^), cortical cross-sectional area (cortical CSA, mm^2^), endosteal and periosteal circumference (EC and PC, mm), total cross-sectional area (total CSA, mm^2^), and bone strength strain index with respect to torsion (polar SSI, mm^3^) were measured using a scan through the diaphysis (at 25% of the bone length in the proximal direction of the distal end of the bone) of the radius and tibia. Trabecular vBMD (mg/cm^3^) was measured using a scan through the metaphysis (at 4% of the bone length) of these bones. Tibia length was measured from the medial malleolus to the medial condyle of the tibia, and length of the forearm was defined as the distance from the olecranon to the ulna styloid process. The CVs were <1% for all pQCT measurements. Due to movement artifacts, bone metal being present or an incorrectly positioned measuring field, two tibia and four radius scans at the follow-up visit were excluded.(19)

### Statistical analysis

All data was analyzed using SPSS software, version 17.0 for Windows (IBM SPSS, Armonk, NY, USA). We used a linear regression model, using continuous variables, with changes in bone parameters as dependent variables and baseline physical activity (h/week) or osteogenic index as well as change in physical activity (h/week) or osteogenic index (between the baseline and follow-up visit) as independent variables, to investigate the association between altered physical activity and changes in bone parameter. For each of the bone variables, which reflected the change, a multiple regression analysis was performed. The change in the bone variable was the dependent variable and the regression model comprised the baseline training (h/week), interaction between baseline physical activity and change of physical activity (Δ physical activity), and, finally, three variables belonging to spline functions of the change of physical activity. The spline functions were linear below the 25 percentile point, quadratic in each of the two intervals from the 25 percentile to the median and from the median to the 75 percentile point, and linear thereafter.

We calculated absolute changes in unadjusted bone parameters as well as amount of physical activity between the baseline and follow-up visits within each group (HH, HL, LH, and LL) using a paired-samples *t* test. Characteristics between subjects divided according to changes in amount of physical activity between baseline and follow-up visit (HH, HL, LH, and LL) were calculated using ANOVA followed by least significant difference post hoc test for continuous variables and χ^2^ test for categorical variables.

To illustrate differences in 5-year change of bone parameters between subjects divided according to changes in the amount of physical activity between baseline and follow-up visits (HH, HL, LH, and LL), delta values for each group at each bone site were calculated. We calculated delta values for each bone parameter as percent differences between baseline and follow-up visits, adjusted for follow-up time. Differences between subjects divided according to changes of physical activity between baseline and follow-up visit (HH, HL, LH, and LL) were calculated on delta values using ANOVA followed by least significant difference post hoc test.

## Results

### Increased physical activity and bone development

**Fig. 1:**
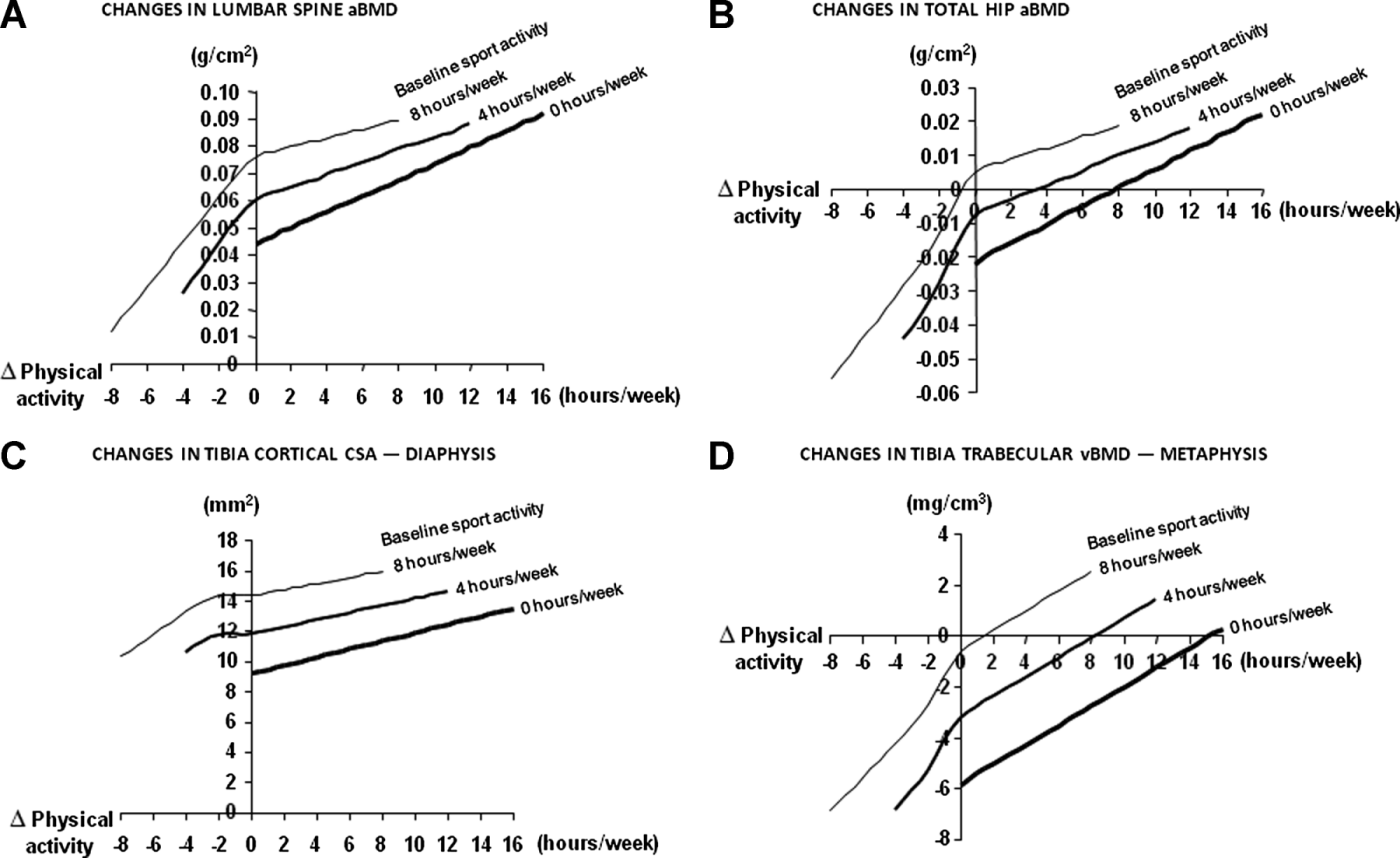
Association between altered physical activity and bone development. Multiple regression analysis with spline functions of the association between changed amount of physical activity in relation to sports (h/week) and changes in (*A*–*D*) bone parameters, based on various amounts of physical activity at baseline visit.

The independent predictive role of change in the amount of physical activity (between the baseline and follow-up visits) as well as baseline physical activity on the change in bone parameters was evaluated using linear regression (Tables 1 and 2). Both increased amount of physical activity between the baseline and follow-up visits, and a high amount of physical activity at the baseline visit, were independently associated with increased BMC of the total body, aBMD and area of the lumbar spine, femoral neck area, as well as of cortical bone size (cross sectional area and periosteal circumference), and bone strength (polar SSI) of the tibia (Table 1). For each hour of increased physical activity, aBMD of the lumbar spine and BMC of the total body increased by 0.005 g/cm^2^ and 5.4 g, respectively, while cortical CSA and total CSA of the tibia increased by 0.36 mm^2^ and 0.49 mm^2^, respectively, between the baseline and follow-up visits (Table 1). These increases occurred from a higher level in men with a high level of baseline physical activity than in men with a low baseline activity, as illustrated in Figure 1. The increased amount of physical activity and amount of physical activity at baseline were also associated with a reduced decrease in aBMD of the total hip as well as in trabecular volumetric BMD at the tibia (Table 1). Similar but weaker associations between change in physical activity and change in cortical CSA as well as trabecular vBMD were found at the radius (Table 2).

**Table 1:** Association Between Physical Activity Change and Change in Weight-Bearing Bone Parameters in Young Men

		Physical activity					
			
		Baseline		Change			
				
	Five-year change^a^ (mean ± SD)	B-absolute	B-relative (%)	B-absolute	B-relative (%)	R^2^ (%)	Osteogenic index change, R^2^ (%)
Measurement site for BMC, aBMD, and bone area using DXA							
Total body BMC (g)^b^	135 ± 160	5.5***	0.16***	5.4**	0.19***	1.2	2.1
Lumbar spine aBMD (g/cm^2^)^b^	0.046 ± 0.061	0.003***	0.24***	0.005***	0.37***	6.0	4.8
Total hip aBMD (g/cm^2^)^b^	–0.023 ± 0.061	0.003***	0.25***	0.004***	0.38***	5.6	5.9
Lumbar spine area (cm^2^)^b^	1.04 ± 1.50	0.03*	0.05	0.08***	0.13***	3.3	3.0
Femoral neck area (cm^2^)^b^	0.089 ± 0.129	0.005***	0.08***	0.005***	0.08***	1.4	–
Measurement site for vBMD, bone size, and bone strength at the tibia using pQCT							
Cortical							
Cross sectional area (mm^2^)^c^	11.30 ± 8.71	0.59***	0.18***	0.36***	0.13***	1.8	2.6
Periosteal circumference (mm)^c^	–0.39 ± 1.01	0.027*	0.04**	0.027**	0.04**	0.8	1.7
Endosteal circumference (mm)^c^	–2.18 ± 1.73	–0.038*	–0.07	–0.005	–0.01	–	–
vBMD (mg/cm^3^)^c^	7.49 ± 13.45	–0.37**	–0.03**	–0.18	–0.02	–	0.6
Polar SSI (mm^3^)^c^	111 ± 63	3.24***	0.13**	1.59*	0.09*	0.7	0.7
Trabecular							
vBMD (mg/cm^3^)^d^	–4.70 ± 14.24	0.55***	0.21***	0.59***	0.22***	1.9	2.6
Total							
Cross sectional area (mm^2^)^c^	–4.16 ± 17.37	0.56**	0.26**	0.49**	0.19*	0.9	2.2

Linear regression model with change in bone parameters as dependent variables, adjusted for follow-up time. Baseline physical activity (h/week) or osteogenic index and physical activity change (h/week) or osteogenic index change between the baseline and follow-up visit were used as independent and continuous variables. Main effects of independent variables are presented as unstandardized coefficients (B). B-absolute denotes B for absolute change in bone variables while B-relative denotes B for percentage change in bone variables per hour change.

BMC = bone mineral content; aBMD = areal bone mineral density; DXA = dual-energy X-ray absorptiometry; vBMD = volumetric bone mineral density; pQCT = peripheral quantitative computed tomography; polar SSI = strength strain index with respect to torsion; R^2^ = percentage of the variation.

a Five-year changes have been reported previously.(19)

b *n* = 828.

c *n* = 832.

d *n* = 831.

* *p* < 0.05.

** *p* < 0.01.

*** *p* < 0.001.

**Table 2:** Association Between Physical Activity Change and Change in Non–Weight-Bearing Bone Parameters in Young Men

		Physical activity			
		
		Baseline		Change	
			
	Five-year change^a^ (mean ± SD)	B-absolute	B-relative (%)	B-absolute	B-relative (%)
Measurement site for aBMD using DXA					
Nondominant radius (g/cm^2^)^b^	0.044 ± 0.022	0.0003	0.02	0.0002	0.04
Measurement site for vBMD, bone size, and bone strength at the radius using pQCT					
Cortical					
Cross sectional area (mm^2^)^c^	2.86 ± 3.47	0.15***	0.15***	0.14***	0.15***
Periosteal circumference (mm)^c^	0.01 ± 0.69	0.012	0.03	0.013	0.03
Endosteal circumference (mm)^c^	–0.58 ± 1.14	–0.018	–0.07	–0.014	–0.06
vBMD (mg/cm^3^)^c^	24.7 ± 15.3	–0.060	0.007	0.028	0.004
Polar SSI (mm^3^)^c^	14.5 ± 16.4	0.71***	0.20***	0.35*	0.13*
Trabecular					
vBMD (mg/cm^3^)^c^	5.85 ± 14.43	0.33*	0.11	0.37*	0.15*

Linear regression model with change in bone parameters as dependent variables, adjusted for follow-up time. Baseline physical activity (h/week) and physical activity change (h/week) between the baseline and follow-up visit were used as independent continuous variables. Main effects of independent variables are presented as unstandardized coefficients (B). B-absolute denotes B for absolute change in bone variables while B-relative denotes B for percentage change in bone variables per hour change.

aBMD = areal bone mineral density; DXA = dual-energy X-ray absorptiometry; vBMD = volumetric bone mineral density; pQCT = peripheral quantitative computed tomography; polar SSI = strength strain index with respect to torsion.

a Five year changes have been reported previously.(19)

b *n* = 832.

c *n* = 829.

* *p* < 0.05.

*** *p* < 0.001.

In order to take variations in baseline level into account, the predictive role of baseline and change in physical activity on relative (%) change in bone parameters was investigated. Using relative change in bone parameters as the outcome variable did not markedly alter the predictive role of baseline and change in physical activity habits (Table 1). The independent predictive role of change in the amount of physical activity (between the baseline and follow-up visits) as well as baseline physical activity on bone parameters was not markedly altered when changes in weight, height, and calcium intake were included as covariates in the linear regression analyses (data not shown). With the aim to elucidate the role of change in body composition on change in bone variables, change in total body lean and fat mass were included (instead of changes in weight) in the regression model. The independent predictive role of change in the amount of physical activity remained but was somewhat weakened concerning change in total body BMC (B = 3.6 (g/h), *p* < 0.05), lumbar spine aBMD and area (B = 0.002 ((g/cm^2^)/h), *p* < 0.001; and B = 0.04 (cm^2^/h), *p* < 0.05, respectively), total hip aBMD (B = 0.002 ((g/cm^2^)/h), *p* < 0.01), femoral neck area (B = 0.003 (cm^2^/h), *p* < 0.05), polar SSI (B = 0.29 (mm^3^/h), *p* < 0.05), and tibial trabecular vBMD (B = 0.4 ((mg/cm^3^)/h), *p* < 0.05). Change in the amount of physical activity did not predict change in bone size of the tibia (cortical and total CSA and cortical PC), when changes in lean and fat mass were included as covariates in the linear regression analyses.

In order to also investigate the predictive role of exercise type (with varying degree of loading), the change in the osteogenic index (between the baseline and follow-up visits) was included in the linear regression model (Table 1) to identify predictors of change in bone variables. Change in the osteogenic index could explain 5.9% and 4.8% of the total variation in aBMD of the hip and spine, respectively. Of the pQCT variables, the osteogenic index explained 2.6% of the variation in cortical CSA and trabecular vBMD, and 2.2% of the total CSA, but only 1.7% of the variation in periosteal circumference (Table 1).

### To illustrate bone development depending on amount of physical activity at baseline and at follow-up

Men were divided into groups based on high (H) and low (L) amount of physical activity at the baseline and follow-up visits.

There were no significant differences in baseline age, weight, height, calcium intake, or smoking prevalence between men in the HH and HL groups or between men in the LL and LH groups (Table 3).

**Table 3:** Characteristics of the Cohort Divided According to Changes of Physical Activity Between the Baseline and Follow-Up Visits

	Changes in physical activity (L < 4 and H ≥ 4 h/week)				
		
	LL	LH	HL	HH	ANOVA *p*
Number of subjects	405	69	213	146	
Baseline					
Age (years)	19.0 ± 0.6	18.9 ± 0.5	18.8 ± 0.5	18.9 ± 0.5	0.084
Height (cm)	181.7 ± 6.5	182.4 ± 7.2	181.3 ± 6.8	181.3 ± 6.9	0.596
Weight (kg)	72.5 ± 12.3	72.2 ± 10.1	75.6 ± 12.6^A^	75.7 ± 9.3^a^	0.002
Lean mass (kg)	55.9 ± 6.0	56.7 ± 5.9	58.8 ± 6.4^A^	60.3 ± 6.0^A^,^B^	<0.001
Fat mass (kg)	13.6 ± 8.4	12.4 ± 6.6	13.7 ± 8.4	12.4 ± 5.9	0.263
Calcium intake (mg/day)	1000 ± 633	1117 ± 765	1240 ± 714^A^	1194 ± 765^a^	<0.001
Smoking (%)	9.6	5.8	6.1	2.7^A^	
Amount of sport activity at baseline visit (h/week)	0.9 ± 1.3	1.5 ± 1.7	7.8 ± 4.0^A^,^B^	9.8 ± 5.6^A,B,C^	<0.001
Follow-up					
Age (years)	24.1 ± 0.6	24.0 ± 0.6	24.0 ± 0.6	24.0 ± 0.6	0.152
Height (cm)	182.2 ± 6.5	183.0 ± 7.3	181.8 ± 6.8	181.8 ± 6.9	0.529
Weight (kg)	77.2 ± 13.2	77.2 ± 9.7	80.1 ± 14.0^a^	80.2 ± 8.6	0.011
Lean mass (kg)	56.9 ± 5.9	60.7 ± 6.3^A^	59.0 ± 6.4^A^	62.5 ± 6.5^A^,^C^	<0.001
Fat mass (kg)	17.5 ± 9.5	14.0 ± 7.0^a^	17.4 ± 8.4^b^	15.1 ± 6.4^a^	0.001
Calcium intake (mg/day)	781 ± 504	868 ± 609	756 ± 493	819 ± 499	0.370
Smoking (%)	10.4	7.2	5.6^a^	1.4^A^,^b^,^c^	
Amount of sport activity at follow-up visit (h/week)	0.8 ± 1.2	6.8 ± 3.2^A,^*	1.5 ± 1.4^A^,^B^,*	7.9 ± 3.9^A^,^b^,^C^,*	<0.001

Values are given as mean ± SD. Differences between the subgroups tested by ANOVA followed by Bonferroni post hoc test for continuous variables and by χ^2^ for categorical variables.

ANOVA = analysis of variance; PA = physical activity; LL = low PA (<4 h/week) at both visits; LH = low PA at baseline and high PA (≥4 h/week) at the follow-up visit; HL = high PA level at the baseline visit but low PA at follow-up; HH = high PA at both visits.

a,b,c *p* < 0.05 for

a vs LL

b vs LH

c vs HL (ie, lowercase a, b, c).

A,B,C *p* < 0.01 for

A vs LL

B vs LH

C vs HL (ie, uppercase, non-bold A, B, C).

A,B,C *p* < 0.001 for

A vs LL

B vs LH

C vs HL (ie, uppercase, bold **A**, **B**, **C**).

* Changes in amount of physical activity between the baseline and follow-up visit within each group (LL, LH, HL, and HH) were calculated using a paired-samples *t* test, *p* < 0.001, *n* = 833.

**Fig. 2:**
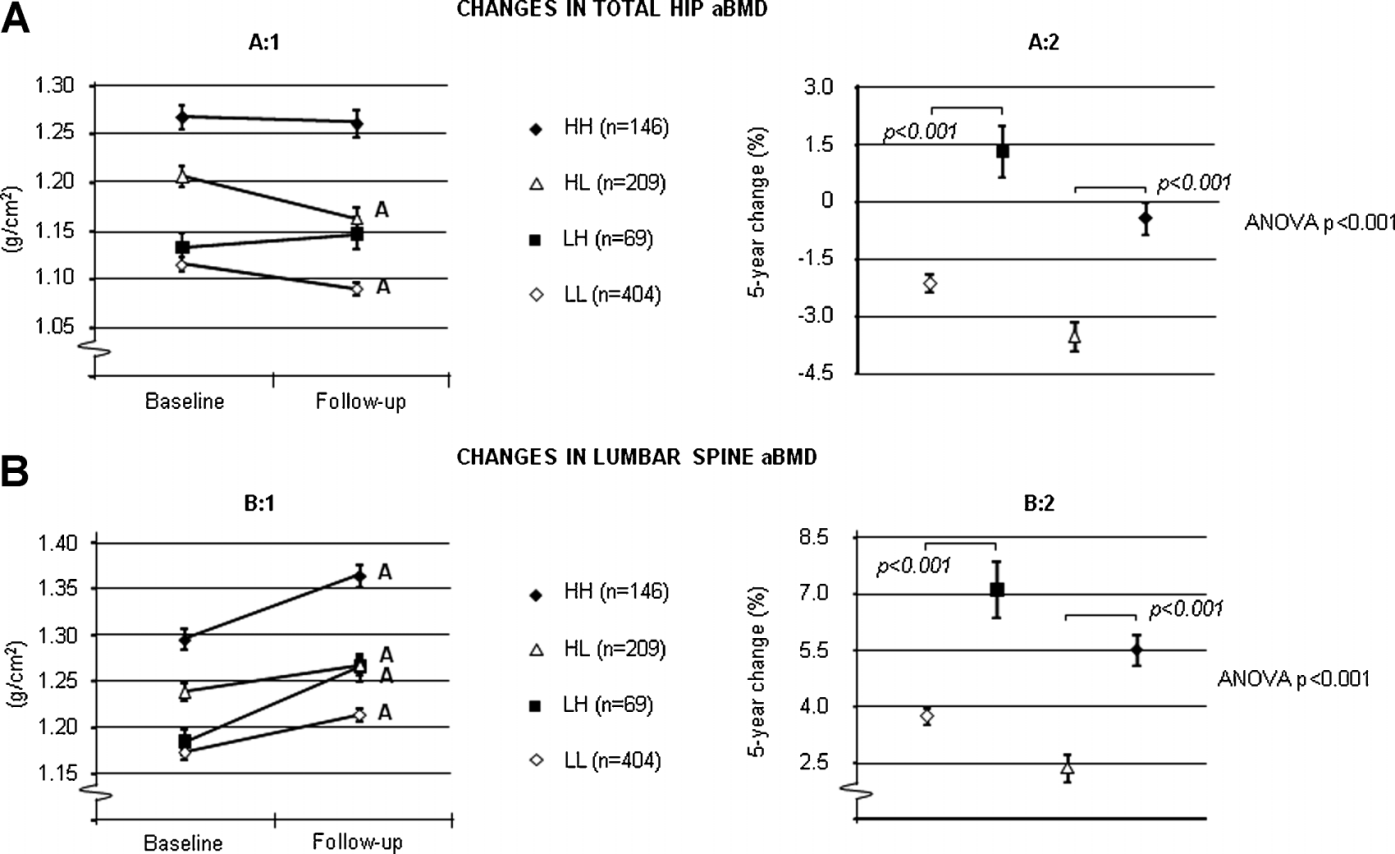
Five-year changes in aBMD of the (*A*) total hip and (*B*) lumbar spine according to changes in physical activity. Consistently high (HH), consistently low (LL), changed from high to low (HL), and changed from low to high (LH) amount of physical activity in relation to sports. Values are given as mean ± SEM. Letters represent *p* < 0.001.

Men in the group who increased their amount of physical activity (LH) gained 1.3% aBMD at the total hip whereas men who remained at a low physical activity level (LL) lost 2.1% aBMD at this site (Fig. 2*A*:1-2). Reduced physical activity (HL) was associated with greater losses in hip aBMD, than a maintained high level of activity (HH) (Fig. 2*A*:1-2). The 5-year increase in aBMD of the lumbar spine was greater in men who increased their physical activity (LH) than men with consistently low activity (LL) (7.1% and 3.7%, respectively; Fig. 2*B*:1-2). Men with a high amount of physical activity (HH) at both visits had a more advantageous bone development at the lumbar spine than men who reduced their activity level (HL) (Fig. 2*B*:1-2).

**Fig. 3:**
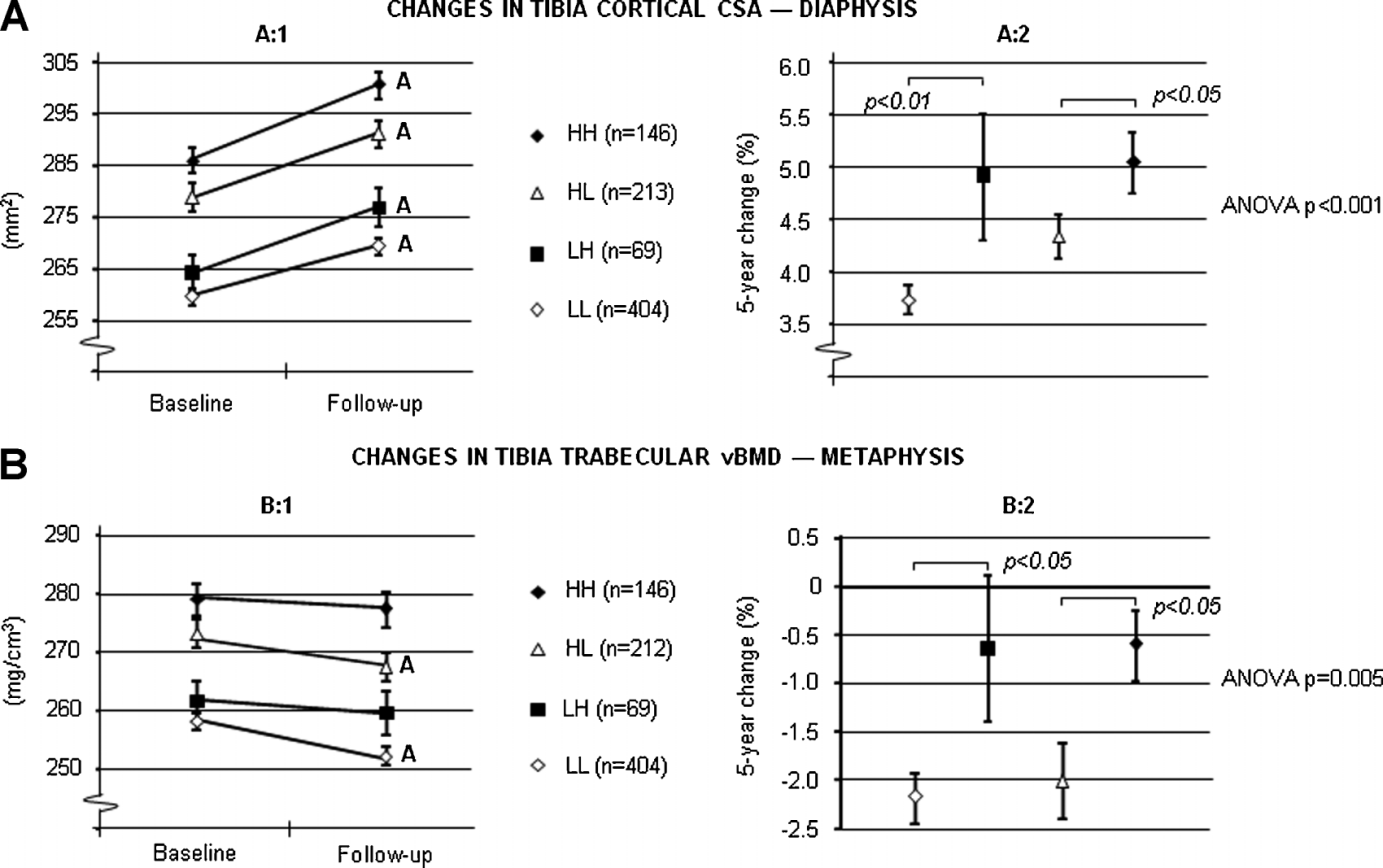
Five-year changes in (*A*) cortical CSA and (*B*) trabecular volumetric BMD of the tibia according to changes in physical activity. Consistently high (HH), consistently low (LL), changed from high to low (HL), and changed from low to high (LH) amount of physical activity in relation to sports. Values are given as mean ± SEM. Letters represent *p* < 0.001.

The 5-year augmentation of the cortical bone size (cross sectional area) of the tibia was larger in men who increased their physical activity (LH) than men who remained at a low amount of physical activity (LL) (4.9% and 3.7%, respectively; Fig. 3*A*:1-2). Reduced physical activity (HL) was related to smaller gains of cortical CSA than a physical activity level that remained high (HH) (Fig. 3*A*:1-2). The 5-year losses in trabecular vBMD at the tibia were lower in men who increased their physical activity (LH) than in men who stayed at a low amount of physical activity (LL) (0.6% and 2.1%, respectively; Fig. 3*B*:1-2). Remaining at a high activity level (HH) was associated with smaller losses at this bone site than reduced physical activity (HL) between visits (Fig. 3*B*:1-2).

## Discussion

To our knowledge, this is the first large longitudinal population-based study investigating physical activity in relation to aBMD and bone structure development in men at the age when peak bone mass is believed to be attained. In the present study, increased physical activity was related to greater gains in aBMD at the lumbar spine and total body. Interestingly, we found that men who increased their amount of physical activity also increased their hip aBMD in contrast to men who remained or reduced their activity level, in whom a reduction in hip aBMD was apparent. Our data indicate that the physical activity accompanied increase in aBMD was due to increased cortical bone size and trabecular vBMD.

Both cortical bone size and BMD are important determinants of bone strength and resistance against fracture.(24, 25) As the resistance of bone to bending and torsion forces is related exponentially to its diameter, even a small difference in the outer circumference could make a substantial contribution to its strength and resistance to fracture.(24) In the present study, we found that increased physical activity was related to augmented cortical bone size (cross sectional area) via actions on the outer cortical envelope (periosteal circumference), indicating attained benefits of physical activity in enlarging the cortical shell, even though physical activity was increased after entering adulthood. Thus, our results suggest that increased physical activity during this age has the ability to prevent the age-dependent decline in aBMD at the hip and increase the cortical envelope, having a positive effect on the attainment of peak bone mass, both in terms of improved cortical structure and in vBMD, in men. We found similar but weaker associations between change in physical activity and change in cortical CSA as well as trabecular vBMD at the radius. We speculate that this finding was due to a much higher degree of loading, by sports activity, exerted on the weight-bearing tibia than on the mostly unloaded radius. In this study, we performed an analysis taking mechanical loading in to account using the osteogenic index, based on ground reaction forces of physical activity. In this analysis, the osteogenic index could explain the change, to a somewhat higher degree than amount of physical activity, in several bone variables, including the cortical CSA and trabecular vBMD. When alterations in lean and fat mass were included as covariates in the linear regression analyses the association between amount of physical activity and bone parameters was weakened or lost, indicating that the effect of change in physical activity on change in bone variables is at least partly mediated via changes in body composition.

The differences in bone parameters according to physical activity behavior during this relatively short period were significant. Hence, the net difference between a man who reduced his activity by 2.5 h/week and a man who increased his activity by 2.5 h/week would equal 0.023 g/cm^2^ for the spine and 0.022 g/cm^2^ for the hip, changes corresponding to 16% and 14%, respectively, of an SD in aBMD.(19) Given that every SD decrease in femoral neck aBMD is associated with approximately a doubled increased hip fracture risk, an alteration dependent on change in physical activity behavior in hip aBMD, as seen in the present study, could result in future increased risk of developing low bone mass and to some extent be of clinical significance.(26, 27)

Trabecular vBMD is thought to be of importance for the structural integrity and strength of the long bones, and has been found to be lower in both young men and postmenopausal women with prevalent fractures than in their nonfracture counterparts.(28, 29) Thus, our data indicate that increased physical activity could have a positive impact on the development of this bone trait, which could have a future impact of fractures associated with trabecular bone loss.

Although our results clearly demonstrate that increased physical activity was associated with greater 5-year gains in aBMD and cortical bone size, it must be emphasized that the men who continued on a high level of physical activity had the highest aBMD and the greatest cortical bone size at the follow-up visit, probably as a result of their long duration and early initiation of physical activity. These findings support previous research indicating the importance of maintenance of a sufficient physical activity to avoid bone loss.(30) In addition, since falling is an even more important risk factor of hip fractures than aBMD,(31) and exercise to maintain physical functioning is the most effective way to reduce the risk of falling,(32) maintenance of physical activity is most likely important at higher ages when hip fractures start to occur.

Using questionnaires to assess physical activity habits is associated with limitations such as obtainment of, to some extent, imprecise physical activity data. In the present study, present sport activity participation was assessed using a self-reporting questionnaire at both the first study visit (baseline) and the 5-year follow-up visit, which limited the risk of inaccurate reporting caused by long time periods of recalling previous physical activity habits.

The use of a measuring technique, pQCT, which enables investigation of the bone geometric properties and the vBMD, together with the large sample size of men followed longitudinally, constitute major strengths of the present study. In addition, the dropout rate was fairly low (22%) and the followed men were not statistically different in anthropometrics and physical activity habits at the baseline visit, suggesting that the longitudinal cohort was representative of the initial population-based cohort. The investigated population, being mainly whites and only males does not allow us to infer that our results will apply to other populations.

In conclusion, this is the first large study reporting that increased physical activity was related to increased aBMD, trabecular vBMD, and augmented cortical bone size in young adulthood, indicating that physical activity during this age has the ability to optimize the attained peak bone mass, which could affect the risk of developing osteoporosis later in life.

## Disclosures

All authors state that they have no conflicts of interest.
